# Sex-Specific Associations Between Sagittal Spinal Alignment and Postural Control in Recreational Strength-Trained Young Adults: A Cross-Sectional Observational Study

**DOI:** 10.3390/healthcare14091156

**Published:** 2026-04-25

**Authors:** Wojciech Kasperek, Fabian Strus, Łukasz Rydzik, Tadeusz Ambroży, Joanna Baran, Maciej Kochman

**Affiliations:** 1 Department of Clinical Physiotherapy in Musculoskeletal Disorders, Institute of Physiotherapy, Faculty of Health Sciences and Psychology, University of Rzeszów, 35-959 Rzeszów, Poland; 2 Department of Sport Theory and Motor Skills, Institute of Sports Sciences, University of Physical Culture, 31-571 Kraków, Poland

**Keywords:** healthcare, posture, biomechanics, physical activity, balance

## Abstract

**Background/Objectives:** Strength training is a widely recommended form of physical activity due to its extensive health benefits and positive effects on musculoskeletal function, although improper technique and balance deficits may increase injury risk. While sex differences in spinal curvature and postural control have been identified in the general population, it remains unclear whether these differences persist among recreationally strength-trained individuals. This cross-sectional study investigated sex-specific differences in sagittal spinal alignment and static balance and examined potential associations between spinal curvature and postural control in trained young adults. The authors hypothesized that sex-related differences would persist despite regular training and that relationships between spinal alignment and balance would demonstrate sex-specific patterns. **Methods**: This cross-sectional study included 124 young adults (59.7% men and 40.3% women). Anthropometric measurements included height, weight, waist circumference, and hip circumference. Sagittal spinal curvature was assessed using an electronic inclinometer, and balance parameters were evaluated using a stabilometric platform under eyes-open and eyes-closed conditions. **Results**: Statistically significant sex-related differences were observed in sacral angle (*p* < 0.001) and lumbar lordosis (*p* = 0.02). Balance assessment revealed significant differences between sexes in several parameters under eyes-open conditions (*p* < 0.05), as well as in mean COP velocity in the anteroposterior direction under eyes-closed conditions (*p* = 0.003). In women, sacral inclination was positively but weakly correlated with selected balance parameters (r = 0.299–0.306, all *p* < 0.05), indicating an association between spinal alignment and postural control. **Conclusions**: The findings indicate sex-specific differences in sagittal spinal curvature and balance, with sacral alignment associated with balance performance in women. Differences in selected balance parameters were also observed independently of spinal curvature. These results highlight the importance of considering sex and spinal biomechanics when assessing postural control in strength-trained individuals and support further research in larger, more diverse populations with varying training experience and age ranges.

## 1. Introduction

Strength training is widely recommended by international organisations as an effective form of physical activity due to its multisystem health benefits [1] including reductions in all-cause mortality, cardiovascular disease and depressive disorders [2]. The American College of Sports Medicine (ACSM) recommends performing resistance training two to three times per week to promote musculoskeletal health [3]. Resistance training involves the execution of muscle contractions against external loads, typically exceeding 65% of one-repetition maximum, and is based on the principle of progressive overload [4,5]. Regular participation induces neuromuscular and structural adaptations, including significant increases in muscle strength and modifications in musculoskeletal function [6].

Although resistance training is generally considered safe, improper technique, insufficient motor control, and impaired postural stability may increase the risk of musculoskeletal overload and injury [7,8]. Postural control and balance play a fundamental role in maintaining biomechanical efficiency during load-bearing tasks. Deficits in balance may contribute to altered movement patterns and increased stress on stabilizing structures, particularly in the lumbar spine and lower extremities [9,10,11]. Moreover, long-term training exposure may lead to structural adaptations in the musculoskeletal system, including changes in sagittal spinal alignment [12,13].

Sex-related differences in spinal curvature and balance have been previously described in general populations; however, the findings are not consistent [14,15]. Some studies suggest that women demonstrate superior static balance, which has been associated with a lower centre of mass and greater joint flexibility [16], while others report poorer balance performance, which may be attributed to lower absolute muscle strength and differences in neuromuscular control strategies [17]. Similarly, sex differences in spinal alignment—such as greater lumbar lordosis in women—are often linked to anatomical and hormonal factors, yet their functional implications remain debated [18,19]. These inconsistencies highlight the complexity of the relationship between biological sex, structure, and motor control. Importantly, the extent to which such sex-related differences persist in individuals engaged in regular resistance training remains unclear. Strength training induces neuromuscular adaptations, including improved motor unit recruitment, intermuscular coordination, and proprioceptive function, which may influence sex-related postural control strategies and spinal alignment [20,21]. However, it remains unclear whether sagittal spinal curvature is associated with static balance performance in recreationally trained men and women, and whether these relationships differ by sex. Investigating potential sex-specific associations between spinal alignment and balance may contribute to a better understanding of structural–functional interactions in physically active adults and help to explain inconsistencies in the literature.

Therefore, the primary aim of this study was to investigate sex-specific differences in sagittal spinal alignment and static balance among recreational strength-trained young adults. The secondary aim was to examine whether sagittal spinal curvature parameters are associated with postural control indices within each sex. We hypothesized that (1) sex-related differences in sagittal alignment would be present despite regular strength training, and (2) the relationship between spinal curvature and balance would demonstrate sex-specific patterns. To our knowledge, this study is one of only a few that have simultaneously examined sex-specific relationships between sagittal spinal alignment and stabilometric balance parameters in recreationally strength-trained adults, providing novel insight into how habitual resistance training may differentially influence postural control and spinal biomechanics in men and women. The study was not designed to evaluate the effect of strength training, but rather to characterize postural and balance features within a trained population.

## 2. Materials and Methods

This cross-sectional study was conducted at Calypso Fitness Club in Rzeszów, Poland, in July 2024. Prior to the research procedures, participants were informed about the study protocol, its objectives, potential risks and their right to withdraw at any time without providing a reason. Written informed consent was obtained from all study participants. The study was conducted in accordance with the Declaration of Helsinki and the protocol was approved by the Bioethics Committee at the University of Rzeszów (no. 013/04/2024).

### 2.1. Participants

Prior to the study, the minimum required sample size was calculated to be 121 participants, assuming a 95% confidence level, a fraction size of 0.9, and a maximum error of 5%. Of 190 invited individuals, 79 were excluded due to not meeting the eligibility criteria. The final sample consisted of 124 strength-trained participants, including 59.7% males and 40.3% females.

The eligibility criteria were: (1) participation in strength training (hypertrophy-strength) at least three times per week for a minimum of one year prior to the study; (2) age between 18 and 35 years; (3) no musculoskeletal injuries or surgeries within six months prior to the study; (4) no diagnosed neurological disorders affecting balance. Participants who did not meet these criteria were excluded. Participants aged 18–35 were included to focus on young adults with fully developed musculoskeletal systems. A minimum of 1 year of strength training was required to ensure adaptation to resistance exercise and avoid including beginners whose spinal curvature and balance may reflect untrained status. Group characteristics are presented in Table 1.

### 2.2. Study Procedures

The study was conducted in July 2024, in the morning before a scheduled training session. Each participant had a minimum 48 h rest period between the assessment and their previous training session. The study procedures included anthropometric measurements, assessment of anteroposterior spinal curvatures, and balance evaluation.

Anthropometric measurements included body height assessed using a Charder HM200M stadiometer (Charder Electronic Co., Ltd., Taichung, Taiwan), (accuracy: 0.1 cm), body weight measured with a medical scale (accuracy: 0.1 kg), and waist and hip circumferences measured using a measuring tape (accuracy: 0.5 cm). Measurements were performed according to the methodology described by Brończyk-Puzoń et al. [22], and the results were used to calculate BMI and describe group characteristics.

Anteroposterior spinal curvature was assessed using an electronic inclinometer application installed on a Xiaomi Mi Note 10 smartphone (Xiaomi Corporation, Beijing, China). Applications of this type are commonly used in scientific research due to their accessibility and feasibility in various environments [23,24,25]. Measurements were performed in a relaxed upright standing position with the lower limbs extended and gaze directed forward [26]. During the examination, the midpoint of the shorter side of the device was positioned at the designated anatomical landmarks for measuring the angles of spinal curvature [27].

The following sagittal spinal inclination angles were measured: the ALFA 1 angle, obtained by placing the upper arm of the inclinometer in the intervertebral space along the line connecting the lower edges of the posterior superior iliac spines; the ALFA 2 angle, measured at the midpoint of the sacrum along the axis defined by the dimples of Venus; the BETA angle, measured at the thoracolumbar junction (Th12-L1); and the GAMMA angle, obtained by positioning the inclinometer at the C7 spinous process. These measurements enabled determination of the lumbosacral angle, lumbar lordosis curvature (KKL), and thoracic kyphosis curvature (KKP). Reference values were 15–30 degrees for the ALFA angle, 30–40 degrees for the KKL, and 30–40 degrees for the KKP [28,29].

Static balance was assessed using the Cosmogamma stabilometric platform (Emildue R50300). A 30 s trial was performed in a relaxed standing position under both eyes-open and eyes-closed conditions. Testing was conducted in a separate quiet room, according to the methodology described by Ćwirlej-Sozańska et al. [30]. The following parameters were recorded: centre of pressure (COP) path length (mm), mean COP velocity (mm/s), mean COP velocity in the anteroposterior and mediolateral directions (mm/s), and COP surface area (Fcop, cm^2^) [31,32].

### 2.3. Statistical Analysis

Statistical analyses were conducted using the Statistica 13.3 software package (StatSoft Poland Inc., Kraków, Poland). Descriptive statistics were calculated for all variables and included the mean, median, minimum, maximum, first and third quartiles (Q1, Q3), and standard deviation. Only height, sacral inclination, thoracic kyphosis curvature, and mean COP velocity in the mediolateral direction were normally distributed; all other variables deviated from normality. Differences between males and females were evaluated using the independent-samples *t*-test or the Mann–Whitney U test, as appropriate. Associations between variables were assessed using Spearman’s rank correlation coefficient. Statistical significance was set as *p* < 0.05.

## 3. Results

The assessment of spinal curvature in the sagittal plane revealed statistically significant differences between sexes for the sacral angle (*p* < 0.001) and lumbar lordosis (*p* = 0.02). No statistically significant differences were observed for thoracic curvature (KKP), indicating a comparable level of this parameter in both groups. Detailed statistical data are presented in Table 2.

Evaluation of balance parameters under the eyes-open condition revealed significant differences between groups. Females demonstrated a greater COP path length (M = 297.54 mm, SD = 83.30) compared to males (M = 269.29 mm, SD = 84.20; *p* = 0.022). Mean COP velocity was also higher in females than in men (*p* = 0.023). Significant differences were observed for mean COP velocity in the anteroposterior direction (*p* = 0.006) and COP surface area (*p* = 0.042). No significant differences were found for mean COP velocity in the mediolateral direction.

In the eyes-closed condition, a statistically significant difference was found for mean COP velocity in the anteroposterior direction (*p* = 0.003), with higher values in females (M = 9.00 mm/s, SD = 2.88) compared to males (M = 7.61 mm/s, SD = 2.99). No other parameters differed significantly between groups, although higher mean values were observed in females across all variables. Detailed results are presented in Table 3.

Analysis of the relationship between balance parameters and spinal curvature revealed a significant positive correlation between sacral angle and COP path length, mean COP velocity, and anteroposterior COP velocity in the female group under the eyes-open condition. This finding indicates greater postural instability with increasing sacral inclination. Correlation analysis of the remaining variables under both eyes-open and eyes-closed conditions showed no statistically significant relationships. Detailed results are presented in Table 4.

## 4. Discussion

The present study examined sex-related differences in sagittal spinal curvature and balance parameters in strength-trained individuals. The main findings indicate that women demonstrated greater sacral inclination and lumbar lordosis compared to men, while no differences were observed in thoracic kyphosis. Additionally, several balance parameters differed between sexes, and correlations between sacral inclination and selected balance parameters were observed in women under eyes-open conditions.

The assessment of spinal curvature angles revealed statistically significant differences between the groups. Females demonstrated a greater mean inclination (22.31°) compared to males (17.51°), as well as higher lumbar lordosis curvature values, with a mean difference of 3.2°. However, both groups remained within generally accepted normative ranges for sagittal spinal curvature. No values exceeded commonly reported normative ranges, suggesting that the observed differences reflect variation within physiological limits rather than pathological deviations. Grabara, in a study involving young adults, also reported greater lumbar lordosis in females compared to men [33]. Similar findings were described by González-Gálvez et al. in adolescents, indicating that boys present smaller lumbar lordosis angles compared to girls [34]. In the present study, no significant differences were observed in thoracic kyphosis between groups. Tuz at al. similarly reported no differences in thoracic kyphosis between sexes while noting increased lumbar lordosis values among women [35]. These findings suggest that sex-related differences in lumbar lordosis may represent a commonly observed morphological characteristic. Ohlendorf et al., in a study aimed at establishing references values for upper body posture, also reported greater lumbar lordosis angles in women [36]. In contrast, Hay at al. suggested that this morphological characteristic may be associated with pregnancy-related adaptations of the female spine [18]. Puszczałowska-Lizis et al. demonstrated that volleyball training may reduce lumbar lordosis in girls aged 10–13 years [37]. Additionally, research conducted on elite Iranian cyclists suggests an increased risk of greater thoracic kyphosis compared to that of non-cyclists [38]. Conversely, Grabara reported that differences in sagittal spinal curvature between athletes and non-athletes may result from long-term exposure to sport-specific training loads [39]. However, these findings across different populations are heterogeneous, suggesting that spinal adaptations may depend on sport type, training load, and developmental stage rather that representing uniform sex-related or training-related effects.

Taken together, the available evidence and the findings of the present study do not indicate deviations from spinal alignment values in strength-trained individuals. However, due to the cross-sectional design and lack of non-trained control group, casual interpretations regarding the effect of strength training cannot be made. Therefore, resistance training appears to be compatible with maintaining physiological spinal alignment and may be associated with favourable musculoskeletal adaptations, although longitudinal studies are required to confirm this relationship. Supporting this, Marcos-Padro et al. demonstrated that outdoor resistance training in middle-aged and older adults has beneficial effects on spinal alignment [40]. Furthermore, a network meta-analysis by Fernández-Rodríguez et al. indicated that strengthening exercises are among the most effective interventions for reducing pain in individuals with chronic low back pain [41]. Similarly, Jorgić et al. emphasized that strength training during childhood and adolescence may contribute to the prevention and treatment of sagittal plane postural defects [42].

In the present study, statistically significant differences in balance parameters were observed between sexes in the eyes-open condition, as well as for mean COP velocity in the eyes-closed condition. These findings suggest that women may experience greater postural instability compared to men under certain testing conditions, particularly when visual input is removed. However, these differences should be interpreted cautiously, as the cross-sectional design does not allow determination of whether they reflect training-induced adaptations or pre-existing sex-related characteristics. In contrast, Sell et al., in a study involving 50 soldiers of both sexes, reported superior balance performance in women [43]. Similarly, Goncalves et al. found that women demonstrated better static balance compared to men [44]. These discrepancies may reflect heterogeneity across studies in terms of participant characteristics, training status, and methodological approaches. Previous studies have often included physically active or military populations, whereas the present study examined strength-trained individuals. Therefore, differences in sample composition and training background may contribute to the inconsistent findings reported in the literature. Additionally, variability in testing protocols, visual conditions, and stabilometric parameters may further limit direct comparability between studies. Improved balance may have important implications for health, particularly with regard to reducing the risk of fall later in life. Sarabon demonstrated that resistance training is effective in improving balance parameters in both young and older adults [45]. Similarly, Thomas et al. reported that physical training, including resistance training alone, is an effective strategy for improving balance and reducing fall risk in older adults [46]. Moreover, Koźlenia and Domaradzki indicated that static balance assessment may serve as a useful tool for evaluating the risk of musculoskeletal injury [47], while Poncumhak et al. demonstrated the usefulness of balance tests in assessing fall risk [48]. These findings are consistent with a systematic review and meta-analysis by Dal Farr et al., which reported that exercise interventions may improve balance in patients with low back pain, although further research is needed to establish clear clinical guidelines [49]. One possible explanation for the lack of significant findings under eyes-closed conditions is that strength-trained individuals may exhibit enhanced proprioceptive control due to neuromuscular adaptations, which may reduce their reliance on visual input for maintaining stable postural control. This interpretation, however, remains speculative in the absence of longitudinal or experimental data.

The present study also identified correlations between sacral alignment and balance parameters in women, specifically COP path length, mean COP velocity, and anteroposterior COP velocity under the eyes-open condition. No correlations were observed in men or in the eyes-closed condition among women. Similar relationships between abnormal sagittal spinal curvature and balance parameters were reported by Żukowski et al. in a study involving children [50]. However, Braczkowska et al., in a study involving young adult men, reported no correlation between lumbar lordosis and balance parameters [51]. In contrast, Sadaghati et al. identified a significant relationship between thoracic kyphosis and postural stability in men but not in women [52]. Additionally, Cha and Park demonstrated associations between spinal biomechanics and balance in patients with low back pain [53]. The absence of similar correlations in men and in eyes-closed conditions suggest that these relationships may be context-dependent and influenced by sensory conditions or sex-specific motor control strategies. These inconsistencies across studies highlight the need for further research and improved standardization of study populations and measurement methods.

From a practical perspective, these findings may be relevant for coaches, physiotherapists, and strength and conditioning specialists working with physically active populations. To our knowledge, this study is among the few to simultaneously examine the relationships between sagittal spinal curvature and stabilometric balance parameters in strength-trained males and females. The main limitations of the present study include the lack of subgroup analyses based on training experience, the relatively narrow age range (18–35 years), the absence of a control group, the lack of verification of proper exercise technique, and the minimum training frequency of three sessions per week, which may limit the generalizability of the findings to pediatric and elderly populations. Additionally, the cross-sectional design precludes any inference about causality between resistance training and the observed spinal or balance adaptations. A further limitation is the anthropometric differences between men and women. We acknowledge that variables such as height, body mass, BMI, and hip and waist circumference may influence both spinal alignment and balance parameters. As these factors were not controlled for, they may have acted as potential confounders. Future studies should address these limitations by including control groups and participants with varying levels of training experience.

## 5. Conclusions

The results provide preliminary evidence of a potential association between spinal curvature and balance parameters. In the female group, sacral alignment was correlated with indicators such as COP path length and velocity, suggesting that spinal biomechanical alignment may influence balance control; however, causality could not be established in this cross-sectional study. Differences in spinal curvature and balance parameters were also observed between males and females. These findings may have potential practical implications for training program design, particularly with regard to highlighting sex-related differences in postural control; however, they require confirmation in controlled or longitudinal studies before specific training recommendations can be made. Further research including a broader range of variables and more diverse study groups is recommended to better understand the relationships between spinal alignment and balance in strength-trained individuals.

## Figures and Tables

**Table 1:** Anthropometric parameters of the study group.

Variable	Sex	Mean	Median	Minimum	Maximum	Q1	Q3	SD	*p*
Age	F	23.84	22.00	18.00	33.00	21.00	29.00	4.70	0.919
	M	23.55	23.00	18.00	33.00	21.00	27.00	4.16	
Height [cm]	F	165.3	165.0	149.0	179.0	162.0	170.0	5.98	<0.001
	M	179.8	180.0	166.0	190.0	176.0	184.0	5.88	
Body weight [kg]	F	60.98	59.00	47.00	90.00	55.00	65.00	8.73	<0.001
	M	79.46	78.50	61.00	105.0	73.00	87.00	9.54	
BMI [kg/m^2^]	F	22.29	21.74	18.21	30.49	20.55	23.41	2.82	<0.001
	M	24.57	24.62	17.82	32.04	22.94	26.04	2.65	
Waist circumference [cm]	F	73.04	70.00	60.00	96.00	68.00	77.00	8.09	<0.001
	M	86.06	87.00	65.00	100.0	81.00	90.00	7.38	
Hip circumference [cm]	F	95.20	94.50	85.00	118.0	90.0	97.0	6.52	<0.001
	M	99.45	100.0	87.0	112.0	96.0	103.0	5.51	

F—female; M—male; Q1—first quartile; Q3—third quartile; SD—standard deviation; *p*—*p*-value.

**Table 2:** Size of spinal curvatures in the study groups.

Variable	Sex	Mean	Median	Minimum	Maximum	Q1	Q3	SD	*p*
Sacral Inclination	F	22.31	22	13	31	20	25	4.49	<0.001
	M	17.51	17	4.3	31	14	21	5.53	
KLL	F	37.26	37.50	18	61	32	41	8.60	0.018
	M	34.06	34.30	22	56	29	39	7.07	
KKP	F	33.38	33	18	49.50	27	39	8.01	0.709
	M	33.96	34	9	57	28.40	40	8.69	

F—female; M—male; Q1—first quartile; Q3—third quartile; SD—standard deviation; *p*—*p*-value; KKL—lumbar lordosis curvature; KKP—thoracic kyphosis curvature.

**Table 3:** Evaluation of balance parameters by sex under eyes-opened and eyes-closed conditions.

Variable	Sex	Mean	Median	Minimum	Maximum	Q1	Q3	SD	*p*
Eyes opened									
COP path length (mm)	F	297.54	278.75	118.24	543.88	247.84	348.14	83.30	0.022
	M	269.29	255.17	143.56	612.77	210.04	303.83	84.20	
Mean COP velocity (mm/s)	F	9.87	9.26	3.94	20.00	8.26	11.34	2.88	0.023
	M	8.93	8.42	4.79	20.42	7.00	10.12	2.82	
Mean COP velocity in the mediolateral direction (mm/s)	F	4.65	4.37	0.97	8.63	3.50	5.50	1.58	0.152
	M	4.35	3.89	1.87	9.67	3.23	4.97	1.71	
Mean COP velocity in the anteroposterior direction (mm/s)	F	6.15	5.75	3.03	15.18	4.83	7.20	2.12	0.006
	M	5.38	4.89	2.70	14.67	4.07	6.30	2.11	
COP surface area (cm^2^)	F	2.00	1.82	0.31	7.32	1.25	2.56	1.29	0.042
	M	1.70	1.39	0.04	11.53	0.85	1.98	1.50	
Eyes closed									
COP path length (mm)	F	404.67	388.24	200.04	680.03	338.03	476.51	107.6	0.091
	M	372.40	362.26	158.56	822.55	287.97	432.10	110.1	
Mean COP velocity (mm/s)	F	13.37	12.57	6.67	22.67	11.05	15.88	3.66	0.116
	M	12.35	12.01	6.19	27.42	9.52	14.40	3.67	
Mean COP velocity in the mediolateral direction (mm/s)	F	6.17	6.10	2.23	11.90	4.20	7.37	2.26	0.839
	M	6.25	6.27	2.30	11.53	4.63	7.77	2.00	
Mean COP velocity in the anteroposterior direction (mm/s)	F	9.00	8.90	3.77	19.47	6.70	10.83	2.88	0.003
	M	7.61	7.00	3.43	21.83	5.50	9.50	2.99	
COP surface area (cm^2^)	F	2.92	2.67	0.81	6.69	2.15	3.82	1.29	0.055
	M	2.60	2.20	0.04	10.37	1.48	3.04	1.63	

F—female; M—male; Q1—first quartile; Q3—third quartile; SD—standard deviation; *p*—*p*-value.

**Table 4:** Assessment of the relationship between balance parameters and spinal curvature.

Variable	Sex	Sacral Inclination		KLL		KKP	
		R	*p*	R	*p*	R	*p*
Eyes open							
COP path length (mm)	F	0.306	0.031	−0.055	0.706	−0.215	0.133
	M	−0.041	0.731	0.132	0.261	−0.064	0.588
Mean COP velocity (mm/s)	F	0.301	0.034	−0.070	0.627	−0.189	0.189
	M	−0.053	0.654	0.153	0.194	−0.050	0.671
Mean COP velocity in the mediolateral direction (mm/s)	F	0.150	0.298	−0.097	0.504	−0.159	0.271
	M	−0.096	0.417	0.155	0.188	−0.081	0.491
Mean COP velocity in the anteroposterior direction (mm/s)	F	0.299	0.035	−0.075	0.604	−0.252	0.077
	M	0.096	0.415	0.097	0.410	−0.073	0.535
COP surface area (cm^2^)	F	0.256	0.073	−0.067	0.646	−0.198	0.169
	M	−0.037	0.753	0.122	0.299	−0.220	0.059
Eyes closed							
COP path length (mm)	F	0.044	0.763	0.051	0.723	−0.08	0.583
	M	−0.022	0.853	0.095	0.422	−0.101	0.390
Mean COP velocity (mm/s)	F	0.053	0.713	0.036	0.804	−0.058	0.69
	M	−0.043	0.713	0.094	0.427	−0.101	0.391
Mean COP velocity in the mediolateral direction (mm/s)	F	−0.079	0.584	0.013	0.929	0.037	0.8
	M	−0.173	0.140	0.16	0.174	0.053	0.654
Mean COP velocity in the anteroposterior direction (mm/s)	F	0.038	0.795	0.045	0.756	−0.11	0.448
	M	0.155	0.189	−0.001	0.993	−0.169	0.149
COP surface area (cm^2^)	F	−0.026	0.856	0.080	0.581	0.015	0.919
	M	−0.008	0.948	0.164	0.163	−0.08	0.498

F—female; M—male; *p*—*p*-value; KKL—lumbar lordosis curvature; KKP—thoracic kyphosis curvature; R—correlation coefficient.

## Data Availability

The datasets used and/or analysed during the current study are available from the corresponding author on reasonable request.
